# Development of ZnO-NPs reinforced chitosan nanofiber mats with improved antibacterial and biocompatibility properties

**DOI:** 10.1038/s41598-025-01669-w

**Published:** 2025-05-13

**Authors:** Parva Safari, Eshagh Zakipour Rahimabadi, Mohammad Reza Vaezi, Aliasghar Behnamghader, Reza Tahergorabi

**Affiliations:** 1 Fisheries Department, Faculty of Natural Resources, University of Guilan, Sowmeh Sara, Guilan 1144 Iran; 2 Research Department of Nanotechnology and Advanced Materials, , Materials and Energy Research Center, Karaj, Iran; 3 Food and Nutritional Sciences Program, North Carolina Agricultural and Technical State University, Greensboro, NC 27411 USA

**Keywords:** Chitosan, Electrospinning, Nanofiber, Optimization, Wound dressing, ZnO-NPs, Nanoscale materials, Nanoparticles

## Abstract

This paper studied the possibility of fabricating a nano-composite based on chitosan incorporated with ZnO-NPs as a promising textile for wound dressing purposes. The nanofiber mat was obtained from dispersions of ZnO-NPs in chitosan-based solution blended with PVA (Cs/PVA/ZnO-NPs scaffold). The extracted chitosan was characterized using FTIR, FE-SEM, XRD, and TGA analysis. The electrospinning optimization process was successfully done for Cs and PVA mixture and a good combination of polymers, solvent, and the ratios developed through an optimization process (10wt.% PVA and 1wt.% CS in AcAcetic 80%). The nanofibers had an average diameter below 200 nm, while the incorporation of ZnO-NPs decreased their average diameter below 150 nm. FTIR, FE-SEM, XRD analysis were used to evaluate the scaffold structure. The FE-SEM analysis proved the smooth and bead-free morphology of the fibers. Elemental analysis of the mat revealed a good distribution of ZnO-NPs along nanofibers. Cell culture studies with L929 mouse fibroblast cells revealed good viability of the cell on the Cs/PVA/ZnO-NPs scaffold. The nanoparticles improved capability of the mat for growth inhibition rate of bacterial colonies and also its wettability. The results also showed the nontoxicity of CS/PVA/ZnO-NPs composite and its considerable potential for future application in wound dressing.

## Introduction

The seafood industry generates a significant amount of by-products which requires fundamental management throughout the production, distribution, and waste disposal chain. By-catch and by-product of aquatic processing make up about 50–70% of the caught or cultured fish and crustaceans, respectively^1^. These discarded substances are rich in biomaterials that are useful for various industrial purposes due to their unique characteristics. Chitosan is one of these biomaterials with unique features and different functions that can be used in many industries^2,3^. It possesses several advantageous properties, including high biodegradability, biocompatibility, and low toxicity. Its multifunctionality arises from amino groups, metal ion binding capacity, and dye removal ability. These characteristics make it particularly suitable for medical and pharmaceutical applications. Notable uses include the fabrication of hydrogels, films, and membranes, as well as scaffolds for the regeneration of bone, cartilage, liver, and other organs in wound healing and tissue engineering. Furthermore, chitosan plays a crucial role in developing synthetic skin matrices, drug encapsulation systems, and coatings for active drug substances, among other applications^4,5^.

Different techniques have been studied to optimize the extraction of chitosan for different purposes such as increasing the yield, reduction of time. Using different organic acids, enzymatic conditions, microwave heating and biological conversion are some of these studies^6,7^. The commercial chitosan nanofibers were obtained from concentrated solvents^8,9^. Chitosan is notoriously difficult to electrospin due to poor solubility and viscosity issues. Therefore, many studies have attempted to combine it with a second polymer such as PLA, PVA, PEO, etc. to stabilize the spinnability of its fibers^10,11^. More recent, the use of nanoparticles in composite fibers in therapeutic applications has been focused on studies^12,13^.

Electrospinning was noticed due to its considerable simplicity, adaptability and potential applications in various fields^14^. Electrospinning is a direct method for producing an ultrathin fiber with diameter scales ranging from micro to nano, which can change the surface morphology of chitosan fiber to make it suitable for wound dressing applications^15,16^. The materials produced in this method have interesting properties for use in various field of medical application such as tissue engineering due to having a diameter similar to the fibers of the extracellular matrix in the body such as collagen^17^. Adding a polymer or secondary compound can help the electrospinning process and increase the biocompatibility of nanofibers^15^.

Reinforcement of wound dressings with metal substances has been the interest of researchers. Metallic nanoparticles have their unique properties including antimicrobial properties, chemical stability, and effective performance to increase the migration of keratinocytes to the wound site due to the release from covering materials which can improve the healing phases of different injuries. ZnO is a nontoxic and multifunctional nanoparticle with biocompatibility and effective antibacterial action that results from a combination of oxidative stress, membrane disruption, ionic interference, and inhibition of protective biofilms. It can promote the scaffold’s hydrophilicity by introducing hydroxyl groups and increasing surface roughness while increasing proliferation and cell attachment by enhancing protein adsorption, ionic signalling, and mimicking natural cellular environments^18-20^. It considered as a multifunctional material and known as GRAS materials that extremely used is various industrial sectors^5^. The well-known mechanism of antibacterial activity of ZnO nanoparticles is the interaction between nanoparticle and cell membrane which disrupts the electrical balance of microorganism cells, exuding the nucleotide content and cytoplasmic liquids leading to the cell rapture and death^21-23^. The literature shows the possibility of obtaining nanocomposite materials based on chitosan with metal oxide nanoparticle such as ZnO and artificial polymers^3,24,25^.

Several studies have explored the development of Cs/PVA/ZnO nanofibrous mats for various biomedical applications. Our research bridges a critical gap by covering the entire process—from the sustainable extraction of chitosan from discarded waste to the optimized electrospinning of a ZnO-NPs reinforced nanofibrous mat. This comprehensive workflow not only enhances material biocompatibility and antibacterial efficiency but also ensures eco-friendly and scalable production, which is rarely addressed in prior works. The objective of this paper is the fabrication of a nanocomposite material based on chitosan incorporated with ZnO-NPs as a promising textile for wound dressing purposes. A key novelty of our study lies in the precise optimization of electrospinning parameters using extracted chitosan. For the reduction of chemical and time of chitosan extraction, the extraction condition was also modified.

## Materials and methods

### Materials

Chemicals and solvents for extraction and electrospinning as NaOH, glacial acetic acid (AcAcid), and hydrochloric acid were obtained from Merck (Merck, Germany). The fresh shrimp (*Penaeus monodon*) shell waste were brought to the laboratory in an ice box from a marine processing plant (Chabahar, Iran).

### Shrimp shell preparation

The fresh shells were washed thoroughly with tap water. After removing the residual blood, viscera, muscles, sand, epiphytes, and other impurities, the shells were boiled for at least 1 h. The shells were drained and subsequently dried in an oven at 80 °C overnight to make the chitin more tenuous and break down its crystalline structure. Dried materials were milled with an electric grinder (Moulinex, France), and turned into a much finer powder by using an electric grinder (Hardstone, GCS2700W, England) under the mesh of < 200 μm, and kept cold in sealed bags for future use.

### Extraction of chitosan

In this study, the chitosan (Cs) extraction method was based on an optimized chemical extraction^26^ with some modifications in the process to improve extraction efficiency and chemical consumption. The extraction was carried out using two methods: the conventional and microwave methods. In conventional method the heating source was a water bath while the microwave technique (BC 320, Butane, Iran) utilized the irradiation heating for deproteinization and deacetylation heating. Inorganic ingredients of the raw materials were dissolved in dilute hydrochloric acid, which preserved the polymer from hydrolysis based on work of Charoenvuttitham and Pratya. (2006)^27^. The treatment was carried out at room temperature under constant stirring at 200 rpm. After filtering the mixture, the residue was neutralized and dried in an oven at 80 °C overnight. The protein content was removed by alkali treatment along with heating the samples during deproteinization according to method of Tolimate et al. (2003)^28^ for changing the medium’s color to a colorless medium (3 h in conventional method and 24 min in microwave-assisted extraction). The filtrate was washed thoroughly to get rid of the excess sodium hydroxide and reduce the acidity toward neutrality. Ultimately, the obtained materials were rinsed with distilled water and dried in an oven overnight at 80 °C. The product in this step was entirely white or very light yellow. Chitin was converted to chitosan in a hot concentrated NaOH solution (1:50)^29^ at an elevated temperature (140 °C) with the microwave oven (600 W/24 min) in three steps and neutralized and in conventional method this reaction was performed during 8–9 h using a water bath (100 °C). The synthesized chitosan was dissolved in diluted acid and freeze-dried to obtain the final product.

### Electrospinning optimization

A single-step electrospinning technique was applied to fabricate the Cs nanofiber and the nanoparticle loading. For electrospinning optimization, the solutions were prepared separately. Cs (1% wt) was dissolved in acetic acid (AcAcid) 50% and 80% (v/v) at room temperature. The solution of PVA was heated up to 70 °C under constant stirring until the solute disappeared completely. Different ratios of Cs: PVA solution was prepared (100:0, 80:20, 60:40, and 50–50). The solutions were blended slowly under constant stirring for several hours. After obtaining complete blending of the polymers, 2 mL of each solution was poured into the syringe and installed inside the electrospinning instrument. The syringe was connected to a stainless steel needle with a gauge number of 22 as the nozzle. The syringe and collector were attached to the anode and cathode poles of the power generator, respectively. The instrument was regulated at a working voltage of 15 kV, a collector distance of 15 cm, and a feeding rate of 0.5 mL/h.

### Synthesis and loading of ZnO-NPs

ZnO-NPs were prepared by the sol-gel method^30^. Zinc dehydrate was added to the 50 mL of solvent with constant stirring to prepare 0.02 M solution. The NaOH 2 M solution was added to the stock solution, drop by drop, under constant stirring until a slight white solution appeared. Then the solution was agitated on a magnetic stirrer for 2 h. The remaining precipitates were washed several times with distilled water to remove the NaOH residue. The precipitated material was then washed with ethanol to remove any impurities. The suspension was centrifuged at 8000 rpm for 20 min. The white retail was dispersed in distilled water twice and sonicated for 10 min. After centrifuging, the final white ZnO-NPs was dried in a hot air oven at 60 °C overnight and kept in polyethylene plastic bags. Cs (1% wt) and PVA (10% wt) were prepared and blended based on the optimized treatment, and then ZnO-NPs (0.5% wt) was added to the solution under constant stirring for at least 1 h. The electrospinning was performed according to the conditions applied before. The nanofiber containing ZnO-NPs was kept sealed until the analysis process.

### Physiochemical properties

The protein and ash contents of raw materials and Cs samples were determined according to AOAC (1990)^31^. The extraction yield was calculated by comparing the weight of the freeze-dried chitosan to the dry raw material. The solubility of chitosan was evaluated based on Rinaudo. (2006)^32^. The viscosity-average molecular weight was calculated by dissolving Cs in the sodium acetate buffer system (0.5 M/AcAcid 0.5 M). The average molecular weight of the sample (Mw) was calculated according to the Mark-Houwink Eq^33^.

### Characterization analysis

The presence of IR bands was the characteristic property of chitosan which was determined by using a spectrophotometer (model 2000, Perkin-Elmer Corporation, USA). Analyses were performed over a 400–4000 cm^– 1^ frequency at a resolution of 4 cm^− 1^. The degree of deacetylation (DDA) of the extracted chitosan was calculated according to Baxter’s Eq. 2^6^. The XRD and crystallinity of the sample were determined by using an X-ray diffractometer (XRD-7000, Shimadzu, Japan), which operated with CuKa radiation at 40 Kv and 40 mA^34^. The measurements were done at 2^θ^=5–40 to obtain XRD graphs. The samples were coated with a thin layer of platinum or gold under vacuum conditions with a spray coating. The SEM (FE-SEM, MIRA3-TESCAN-XMU, Czech Republic) device verified the sample’s morphological analysis. The average diameters of the nanomaterials were measured based on fifty numbers of nanofiber and nanoparticles that were selected at random and quantified by using Image J analysis software (Image J 1.52 p., USA). The operating voltage was regulated at 20 kV and the images were taken with different regions and magnifications. X-ray energy diffraction spectroscopy (EDS) was performed to plot the distribution of oxidized nanoparticles on the electrospun nanofibers. Point analysis was performed using an X-ray energy analyzer connected to the FE-SEM device. Continuous scans were performed in 2θ ranging 5–60 degrees at fixed intervals of 0.02 at a voltage of 40 kV and a current of 40 mA. The tensile strength of the composite (piece 1 × 10 cm) was evaluated according to the standard ASTM D412, using a universal device (Universal Testing Machine- model/STM-50, Santam Co). The gauge length was regulated at 20 mm.

The wetting test was carried out using a CAM (contact angle measuring) device developed in the Wetting and Fluids Laboratory (Materials and Energy Research Center, Karaj, Iran). The image was recorded using a DFK 23U618USB 3,0 color industrial camera with the help of a 2X lens. Ultra-high pure water was used to measure the contact angle at room temperature. The thermal properties were performed through thermo-gravimetric analysis in an alumina cell using a TGA instrument (TG 209F3 NETZSCH, Germany) by taking a sample of 1 to 2 mg. The measurement was done at a rate of 10 °C/min (under N) from room temperature to 800 °C.

### Antibacterial assay

The antimicrobial performance of the Cs and the Cs/PVA/ZnO-NPs composite against *Escherichia coli* (Gram-negative) and *Staphylococcus aureus* (Gram-positive) bacteria was measured by using the viable cell counting method^35^. Briefly, a suspension (100 µL) with a concentration equivalent to 0.5 McFarland (1.5 × 10^8^ CFU/mL) was prepared from a fresh culture of bacteria. After that, several decimal dilutions were done until the concentration of bacteria reached 1.5 × 10^6^ CFU/mL. The Cs and Cs/PVA/ZnO-NPs composite were exposed to the bacterial nutrient solution for 24 h. An aliquot (100 µL) was taken and put into the sterile plates, and then 15 mL of nutrient agar was added to the plate and shaken gently. The cultures were placed in an incubator at 37 °C for 48 h. After culturing, the surviving population was calculated based on the number of colonies compared to the control. Data were brought as the mean of replicates.

### Cell viability assay

In vitro assessment of biocompatibility and cytotoxicity of the Cs and Cs/ PVA/ZnO-NPs was performed by cell culture and MTT test. An experiment was conducted on the L929 cell, which was a sub clone of parental strain L derived from normal subcutaneous areolar and adipose tissue of 100 day-old male C3H/An mouse, to investigate the cytotoxic effect of the polymer and the nanocomposite (ISO 10993-5). L929 cells were held separately in DMEM (containing 10% FBS, 1% streptomycin-penicillin, and 0.2% gentamycin). The cultured cells were separated by enzyme solution (trypsin 0.25% w/v) and were seeded on 96-well plates and incubated in a CO_2_ Incubator (5% CO_2_, 37 °C). The sterile sample (100 µL) was added to the cultured cells, cytotoxicity was evaluated after 72 h, and the cell viability was determined by 3-(4, 5-dimethylthiazol-2-yl)-2, 5-diphenyl-tetrazolium bromide (MTT) assay at 570 nm using a micro plate reader.

### Statistical analysis

Experiments were designed in triplicate. All data were analyzed using IBM SPSS Statistics (V 26.0.), and the Mean values were compared according to ANOVA analysis. Striking differences were determined with Duncan’s test at a probability level of < 0.05.

## Results and discussion

### Physicochemical properties

The results of the chemical composition of the shell waste of *P. monodon* are presented in Table 1. The ash content of dry raw materials and chitosan was 23.84% and 0.26%, respectively. The moisture content of the samples showed that the conversion of raw materials into dry powder reduced the humidity from 85.60% in fresh shrimp shells to 68.77% and 0.00% in dry powder and chitin, respectively. Shrimp shells contain hydrophilic compounds such as proteins and fats that can absorb moisture and retain moisture in the powder. Also, the cellular structure and texture of the shells have microscopic pores that can retain moisture. These factors cause shrimp shell powder to remain high in moisture even after the drying process. In the study of Younes et al. (2016)^36^, the moisture content of the homogenous waste of *Metapeneaus monoceros* shrimp was 88%. The shrimp *Pandalus borealis* shells contain high amounts of 46.10% ash by dry weight^37^. Based on studies, the source used for chitin extraction can affect the percentage of chemical components, especially the amount of ash content^34^. Like other crustaceans, the main mineral found in shrimp cuticles is CaCO_3_ in the form of aragonite, which gives structural resistance to the skeleton of the organism through Ca^2+^ deposition for environmental survival^1^. Also, the phase of physiological growth and seasonal changes can affect the qualitative and quantitative aspects of the chemical composition^28^. It can explain the high ash contents of such species. In the present study, multiple treatments in the purification process were effective according to proximate analysis (Table 1). This approach exposed the reaction substances to the digestion medium and provided good accessibility during repeating processes. The number of baths for demineralization depends on the species and source of raw materials^34,38^. Also, achieving better solubility of the chitosan can be associated with low amounts of ash^39^. It is said that the commercial chitosan has an ash content of less than 1%, which is similar to the ash content of the chitosan prepared in the present study^26^.

**Table 1:** Physicochemical composition and characterization of Raw materials and purified Chitosan from *P. monodon* (Conventional and microwave-assisted methods).

Physicochemical characterization	Raw shell	Conventional extraction (8–9 h)	Microwave-assisted extraction (24 min)
Ash (%)	23.84 ± 0.78	0.28 ± 0.16^a^	0.26 ± 0.05^a^
Yield * (%)	---	34. 90 ± 0.92^a^	35.13 ± 0.69^a^
Degree of deacetylation (%)	---	84.19 ± 1.95^a^	82.87 ± 4.36^a^
Solubility (%)	---	99.99 ± 0.01^a^	99.99 ± 0.01^a^
Molecular weight (kDa)	---	203.70	146.00
Index of crystallinity (%)	---	35.38 ± 1.25^a^	37.06 ± 0.55^a^

^*^ Based on dry shrimp shells to chitosan.

-Data are the mean ± SD of *n* = 3.

^−^Different letters indicate a significant difference in raws, p< (0.05).

Chitosan manufactures usually need high temperatures and chemicals in large quantities and it takes much time and consumes a lot of energy where will give bad effect to the environment. Microwave irradiation is known to heat materials more efficiently compared to conventional method and chemical reactions process will occur faster by microwave irradiation^40^. Cheng et al. 2020^41^ compared the physicochemical properties of chitosan prepared by microwave and water bath heating with an equivalent quantity of heat intake. Their results revealed that there was no significant difference in the structure of chitosan produced by the two heat sources. The results showed that chitosan successfully prepared by microwave heating within 60 min, while a longer time of 180 min was required for the preparation of chitosan using the conventional heating method under the same heating rate. Even under the same temperature conditions, microwave technology can greatly reduce the reaction time by approximately 1/3. Their results showed that microwaves may efficiently promote complete chemical reactions by the friction heating mechanism generated by molecular vibration beyond a rapid heating source, turning into a more efficient, energy-saving, and environmentally friendly method for the further use of rigid shrimp shells and highly crystalline crustacean materials as evidenced by the comparison of the two extraction methods in the present study. During the deproteinization of chitosan, the decolorization also occurred in 24 min by the microwave heating method, simultaneously. The white appearance of the chitosan showed that the demineralization and deproteinization processes were effective even without the decolorization step (which is shown in a graphical image). As a concept of “green chemistry”, the chemicals of demineralization and deproteinization were considered very mild compared to other studies, and microwave heating reduced the time, chemicals, and steps. Recently microwave irradiation as nonconventional energy sources is widely used in chemical reactions. It was stated that chitin conversion to chitosan by microwave heating was more efficient than the conventional heating. This technology can save huge amount of energy when implemented on industrial scale and is a highly economical extraction technique^42^. In study by Mahdy Samar et al. 2013^43^, chitosan is produced from shrimp waste under microwave irradiation. The process described a rapid synthesis procedure in comparison to conventional methods. It was stated, the microwave technique can be very useful for synthesizing good functional properties chitosan with rapid and clean chemistry. Also, El Knidri et al. 2019^42^ found that using microwave heating for 10 min resulted high degree of deacetylation, compared to conventional heating, which required over several hours for similar characteristics. By this technology, they have successfully produced the chitosan with low alkali concentration, which allowed to avoid manipulating an extremely high concentration of a corrosive substance, especially at industrial scale.

In this study, the products exhibited similar characteristics, leading to comparable outcomes in both extraction methods. This demonstrates the efficacy of the modified extraction method. Furthermore, replacing conventional heating with microwave irradiation in chitosan production achieved similar physicochemical properties and completing the process (Table 1).

### Degree of deacetylation (DDA) and molecular weight (MW)

The DDA is a critical factor that influences physicochemical and biological characteristics such as electrostatic, biodegradability, chelating ability, and electrostatic properties^44,45^. Acetyl groups are removed fully or partially by immersion of the chitin in a hot concentrated alkali solution (100 °C–100 °C < ) for at least 30 min to 8–9 h^46^. Based on the literature, deacetylation occurs more rapidly in the first minutes of the process. In microwave-assisted extraction, this reaction can reach more than 80% only after several minutes of irradiation. In this study, the DDA of the synthesized chitosan was determined at 82.87% according to the ratio of the bands at A_1655_ cm^− 1^ and A_3450_ cm^− 1^ based on the Baxter equation (Table 1). In comparison to the conventional method, in which the deacetylation reaction occurs slowly, microwave irradiation can raise the reaction temperature rapidly by linking solution molecules directly. Mohanasrinivasan et al. (2014)^39^ reported that the DDA of the chitosan (from the shrimp waste of local markets) was 72.82%. Kumari et al. (2017)^33^ also reported 78% DDA for chitosan extracted from *Crangon crangon* shrimp. Differences in extraction conditions and deacetylation processes can account for the varying degrees of deacetylation observed in different studies. The quality of chitosan also depends on its DDA which will also affect the polymer’s solubility. The acetyl groups are firmly attached to chitin, making it difficult to eliminate them through polysaccharide hydrolysis. This process requires harsh alkaline methods to remove acetyl groups placed on the back region of a complete amino group (-NH_2_). Therefore, it requires high sodium/potassium hydroxide concentration, high temperatures, and appropriate time. Numerous published studies report that chitosan extraction with a higher degree of deacetylation requires a sodium hydroxide concentration of about 60% ^29,32,47^. The lower solubility of chitosan can be due to the insufficiency of deacetylation. This issue indicates that the dissolution of chitosan is dependent on the removal of acetyl groups in the deacetylation process, and a lower amount of deacetylation can have adverse interference effects on its performance^43,48,49^. The viscosity-dependent Mw of the chitosan, which is critical affective parameter on the different physicochemical properties of chitosan, was 146.00 kDa. According to the reports, the average Mw is determined by the DDA, source, extraction method, and solution properties^33^.

### FTIR and XRD characterization

**Fig. 1:**
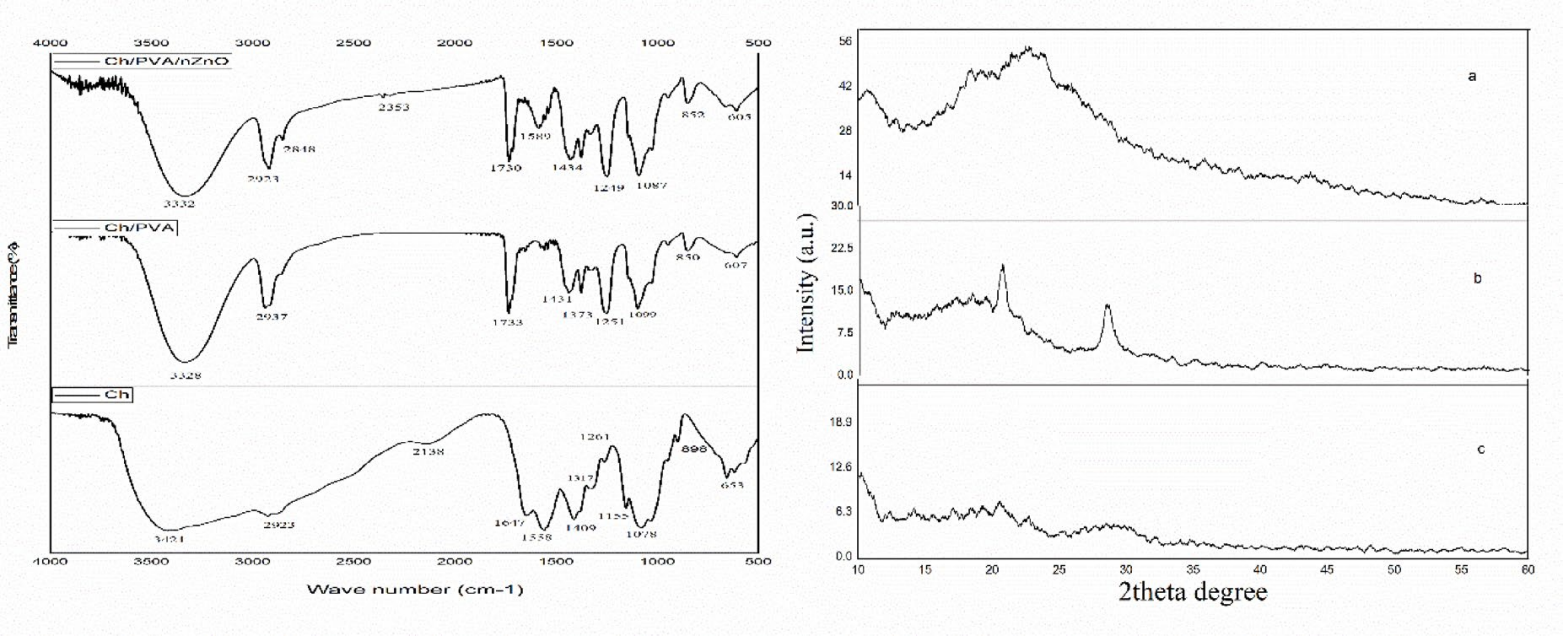
FTIR spectra and XRD pattern of Cs (**a**), Cs/PVA (**b**), and Cs/PVA/ZnO-NPs (**c**).

The FTIR spectra verified the synthesis of chitosan (Fig. 1). The main characteristic bands of chitosan correspond to the vibrations of its functional groups, including hydroxyl (-OH), amine (-NH₂), amide (C = O), and ether (C-O-C) bonds that appeared at around 3400 –3300 cm^− 1^ (broad band due to hydrogen bonding) are related to –NH_2_ and –OH stretching. The C-H stretching was appeared at 2960 –2870 cm^− 1^. In addition, the reduction of hydrogen interactions and free hydroxyl groups is due to the removal of acetyl groups which reduced the NH_2_ bending vibration in the amino group (1593 cm^− 1^), C-O stretching in acetamide (1010 cm^− 1^), 1655 cm^− 1^ (amide I), 1580 cm^− 1^ (NH_2_- bending) and 1320 cm^− 1^ (Amide III). The formation of chitosan after the deacetylation process caused bands to appear at two peaks, 1624 cm^− 1^ and 1403 cm^− 1^^50–52^. Absorption bands at 1160 cm^− 1^ (an asymmetric stretch of C-O-C bridge) which was due to ether bonds in polysaccharide backbone of chitosan. Also the band around 1082 cm^− 1^ and 1032 cm^− 1^ are vibration bending including C-O stretch are characteristic of saccharide structure^51,52^. It is stated that reduction at the band at 1620 cm^− 1^ (NH bending vibration in the R-NH_2_ group) indicates an increase in the DDA, and the absence of bands at 1540 cm^− 1^ proves the efficiency of deproteinization and the purity of the extracted chitosan^1,52^. In the current study, FTIR spectroscopy confirmed the functional groups of chitosan in the modified extraction. The modified (microwave-assisted) extraction process used in this study showed similar crystallinity percentages compared to studies that used conventional methods^34^. This indicates that similar results are achieved despite the significant time savings achieved with microwaves^6^. The XRD analysis identified the structure of chitosan extracted from *P. monodon*. Also, Table 1 represents the relative crystallization index of the extracted chitosan. Following the diffraction pattern of chitosan in Fig. 1, the sample shows the characteristic peaks of chitosan at 2^θ^ of 9–10 ° and 21–22 ° which refers to the presence of hydroxyl and amine groups of the polymers and confirms the formation of chitosan in the modified extraction method. Nouri et al. (2016)^53^ showed chitosan characteristic peaks at 2^θ^ of 11.7°, 22.3 ° and 35° which is due to different sources of extraction. Hajji et al. (2014)^34^ reported that the reflection at 2^θ^ of around 9–10 ° is lower than the peak of around 20°. It could be due to the effect of water molecules and intramolecular hydrogen bonds that decrease after the deacetylation in α-chitin and lead to a more amorphous state in the end products. The reflection at 30° is related to mineral contents, and this peak is observed to be very small due to the low amount of mineral content in the sample of the present study. Chitosan is an amorphous polymer, and the lower sharpness of the peak confirms its lower crystallinity (Table 1). The reflection peaks at 2^θ^ of about 10^o^ and 20^o^ are the fingerprints of the semi-crystalline structure of the chitosan^39^.

### Scanning electron microscopy (SEM) and thermal gravimetric analysis (TGA-DTA)

**Fig. 2:**
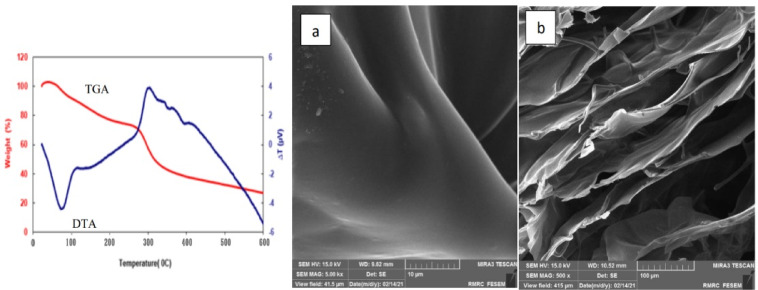
FE-SEM images: surface action (**a**), cross-section (**b**), and TGA-DTA thermogram of chitosan extracted from shrimp shell wastes.

SEM analysis evaluated the morphological characteristics of the chitosan. Microscopic images showed that the synthesized chitosan was smooth and integrated completely. The chitosan sample had a very smooth and flake-like appearance. These results indicate the efficient removal of minerals and proteins according to the modified method. Srinivasan et al. (2018)^54^ reported fibrous structures for chitin and chitosan. Also, Kucukgulmez et al. (2011)^55^ showed a dense, layered, and porous structure for chitosan extracted from the shell of the shrimp *Metapenaeus stebbingi*. The higher magnifications showed the fibrous and layered structure of chitosan. The shrimp, *Liptopenaeus vanamei* showed a similar form in the reports^56^. Thermal stability provides valuable information about thermal stability and structural changes compared to the original sample. In a structure with less crystallinity, thermal decomposition occurs at lower temperatures. TGA was performed at a temperature ranging between 25 °C and 600 °C. As shown in Fig. 2, the TGA curve represents weight loss in two stages. The first occurred around 70–80 °C (~ 75 °C), and the second was at 300–310 °C (~ 305 °C). The first stage usually takes place in the range of 50–120 °C because of the evaporation of the water molecule, which is about 10% of the initial weight of the sample^33^. This layer of water molecules makes the polymer hydrate, and the polymer is intended to absorb a high amount of this kind of water efficiently. The second stage occurred at 390–500 °C, when about 60% of the sample disappeared. Thermal decomposition and breakdown of the glycosidic bonds of the chitosan take place, which reveals the thermal stability of the polymer^57^. TGA analysis confirmed a considerable agreement between the decomposition temperatures of the extracted chitosan in this study and the standard range mentioned in other studies (200 °C to 350 °C)^58^. As seen in Fig. 2, the result of the DTA analysis was inconsistent with the TGA curve. The first endothermic peak is related to moisture remaining in the sample that has not been removed entirely during drying. The second exothermic peak showed the polymer thermal degradation which was confirmed by the TGA curve. The TGA investigated the thermal behavior of the chitosan and reaction stoichiometry. Also, according to documents, the thermal degradation of chitin and chitosan appears at a temperature of about 372 °C and 300–303 °C, respectively. It is because of that chitin has higher heat resistance than chitosan due to its more tenuous and rigid crystalline structure^49,59^.

### The electrospinning optimization of Cs/PVA

**Fig. 3:**
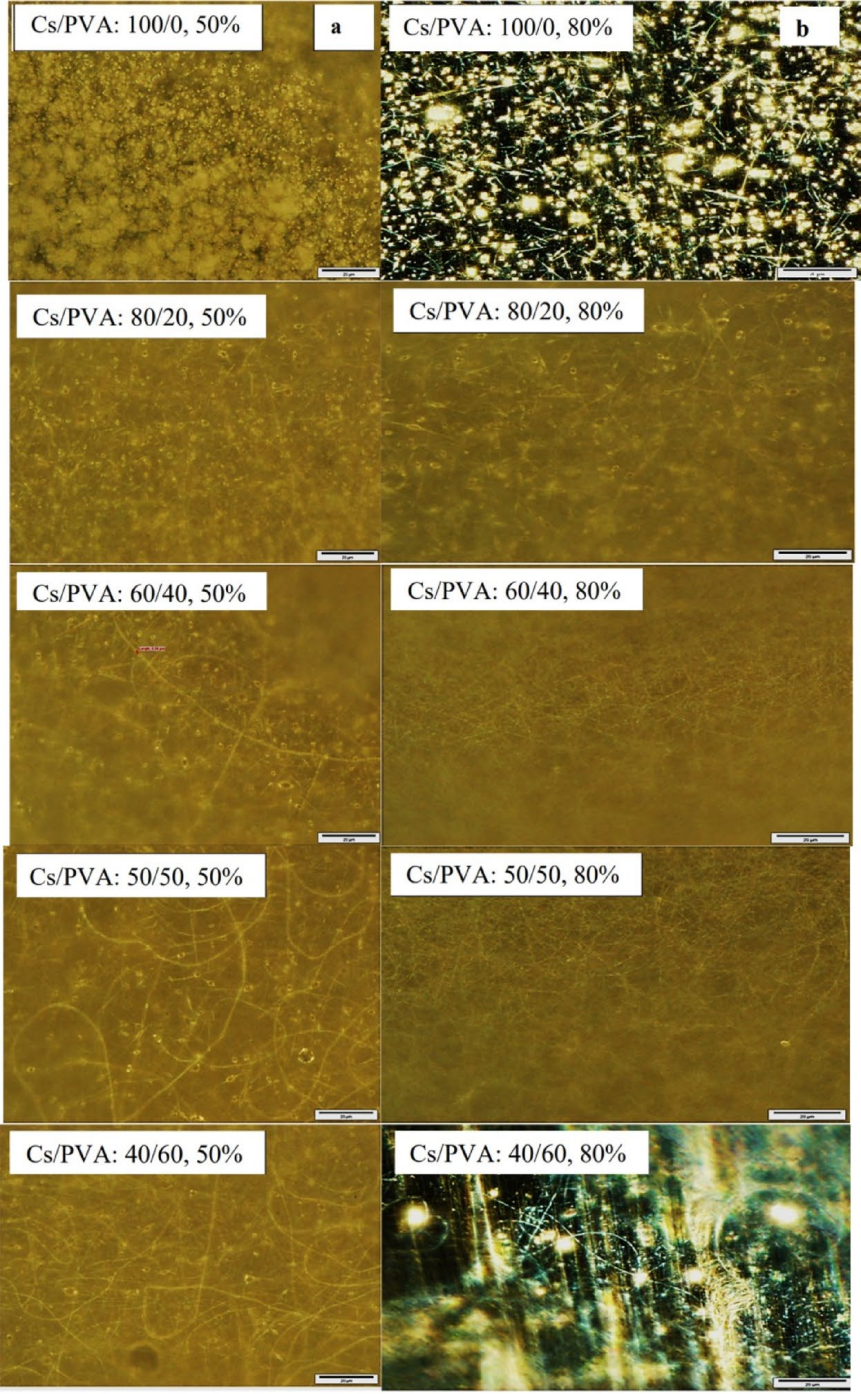
Inverted light microscope images of optimization process of Cs/PVA electrospinning with different ratios and AcAcid concentrations in different magnifications (10x, 20x, and 50x), column (**a**) AcAcid 50% and column (**b**) AcAcid 80%.

The electrospun nanofibers were first evaluated with an inverted light microscope. The microscopic images showed the result of the morphological investigation of the nanofibers in the different mixtures of polymers (Cs/PVA) in AcAcid 50 and 80% v/v (Fig. 3a, b). According to the results, with the increase in the chitosan percentage in the composite, the rate of continuous and bead-free fibers decreased. The results showed that at an acid concentration of 50%, the nanofiber showed a high rate of beading in different ratios of Cs/PVA (Fig. 3a). In this acid concentration, the more appropriate ratio of Cs/PVA was 40:60. In the next step, increase the acid concentration to 80%, making it possible to prepare quality nanofibers with a higher ratio of Cs. As depicted in Fig. 3b, with increasing acid concentration and PVA participation, the electrospinning process showed better conditions in fiber quality in contrast to pure Cs and low acid concentration (Fig. 3b). The morphological study of nanofibers in different treatments showed that at higher concentrations of AcAcid, the fiber generation was continuous and bead-free with elongation and uniformity, and the acid concentration was a very significant factor in the optimization process. The results reveal that the Cs/PVA in ratios of 60:40, 50:50, and 40:60 reached a stable condition and appropriate morphological characteristics, so the treatment with a higher amount of Cs along with quality fibers was used for nanoparticle loading. The diameter distribution of the fibers is brought next to each image, separately. Electrospinning of chitosan and other natural polymers is a complex process due to the particular behavior of the solution, polycationic characteristics, and wide range of the polymer molecular weight. Because of the polycationic nature of chitosan, its electrospinning process faces various challenges and usually needs toxic and strong solvents.

Researchers have used other polymers including polyvinyl pyrrolidone, polyethylene oxide, polyvinyl alcohol, and etc., to overcome the problems of chitosan electrospinning^60-62^. PVA, a biodegradable polymer, can stabilize chitosan for electrospinning and reduce the crystallinity of the polymer^4^. PVA is also one of the synthetic polymers used in many studies to produce composite nanofibers with superior mechanical properties^35^. Based on the microscopic images of nanofibers, acid concentration demonstrates an evident effect on fiber quality. Therefore, at 50% AcAcid a higher proportion of PVA is required to form continuous and bead-free fibers. The amine groups of the chain, the nature of the polymer, high viscosity, and repulsion force among polycations are the main problems that prevent the pure polymer from electrospinning. The second polymer plays a very vital role in smooth fiber formation and continuous quality through the hydrogen bonds between the functional groups of the involved polymers^50,63^. Improper proportions of polymer and solvent lead to an interrupted nanofibrous structure. The high rate of bead formation and inconsistency in fiber morphology were the optimization challenges for achieving the correct combination of polymers^63^. When the contribution of chitosan is higher than that of the second polymer, the fiber formation tends to have a semi-continuous and bead-like behavior. The semi-fibrous structures with bead scattering and the solution drop spraying evaluated the fiber as unacceptable. Also, at low concentrations of AcAcid (50% or less), the formation of nanofibers was more inexpedient, because the amine groups of chitosan are prolonged in solution and create a more viscous solution that eventually requires a higher voltage for spinning.

According to Sarhan and Azzazy (2015)^62^, a low concentration of AcAcid with increasing solution viscosity makes fiber formation a challenge. Under the optimized conditions, increasing the concentration of acetic acid up to 80% v/v, the nanofibers were in circular form and had smooth surfaces with the same diameter along the fiber length. The optimized treatment creates appropriate viscosity, proper interaction, and engagement of the polymer chains. In a study by Ohkawa et al. (2004)^64^, the morphology of chitosan fibers depends on TFA acid concentration. According to their investigations, there are two reasons for the successful electrospinning of chitosan using TFA acid. First, salt formation occurs through engagement with the amino groups of chitosan and the destruction of the strong interactions between chitosan molecules during electrospinning. The second is the volatility of TFA acid and rapid solidification of the chitosan-spinning jet. This feature provides the possibility of electrospinning the higher weight% of pure chitosan. Abdelgawad et al. (2014)^65^ stated that a high percentage of PVA (above 70%) is a necessity to produce uniform fibers of chitosan, and increasing the chitosan (above 40%) decreased the mat quality. Also, when the amount of chitosan increases by more than 50% of the total, it is hard to achieve quality fibers.

These results reveal that when the concentration of chitosan as a poly-cation increases in solution, the repulsive forces between the cationic groups of the structural skeleton increase and prevent the formation of continuous fibers. These results are generally inconsistent with the present study, but the stabilization of chitosan/PVA nanofiber was recorded at a ratio of 60:40 under the optimized condition, in which the formation of healthy fibers was completely confirmed. The lower surface tension of the solution helps the electrospinning process occur in a lower electric field, and the concentration of acetic acid strongly affects the surface tension of the chitosan solution. Therefore, the chitosan dissolution in the strong acetic acid can elucidate the main problem of its electrospinning by causing a decrease in surface tension and an increase in the charge density of the solution. Also, finding the appropriate polymer concentration leads to jet formation simultaneously^8^. The PVA cooperative role involves reducing the viscosity of the solution through the reactions between chitosan and PVA chains. In these reactions, the chain functional groups are involved in secondary bonds that can decrease the solution viscosity^50^. Among the parameters, acid concentration has been reported as the most critical parameter in chitosan electrospinning^8^. Hadjianfar et al. (2019)^66^ stated that the amount of chitosan in the composite has a noteworthy consequence on fiber diameter. Shalumon et al. (2011)^30^ reported an average diameter of about 190–240 nm for the obtained fibers of sodium alginate/PVA.

### The optimized treatment loading and elemental analysis

**Fig. 4:**
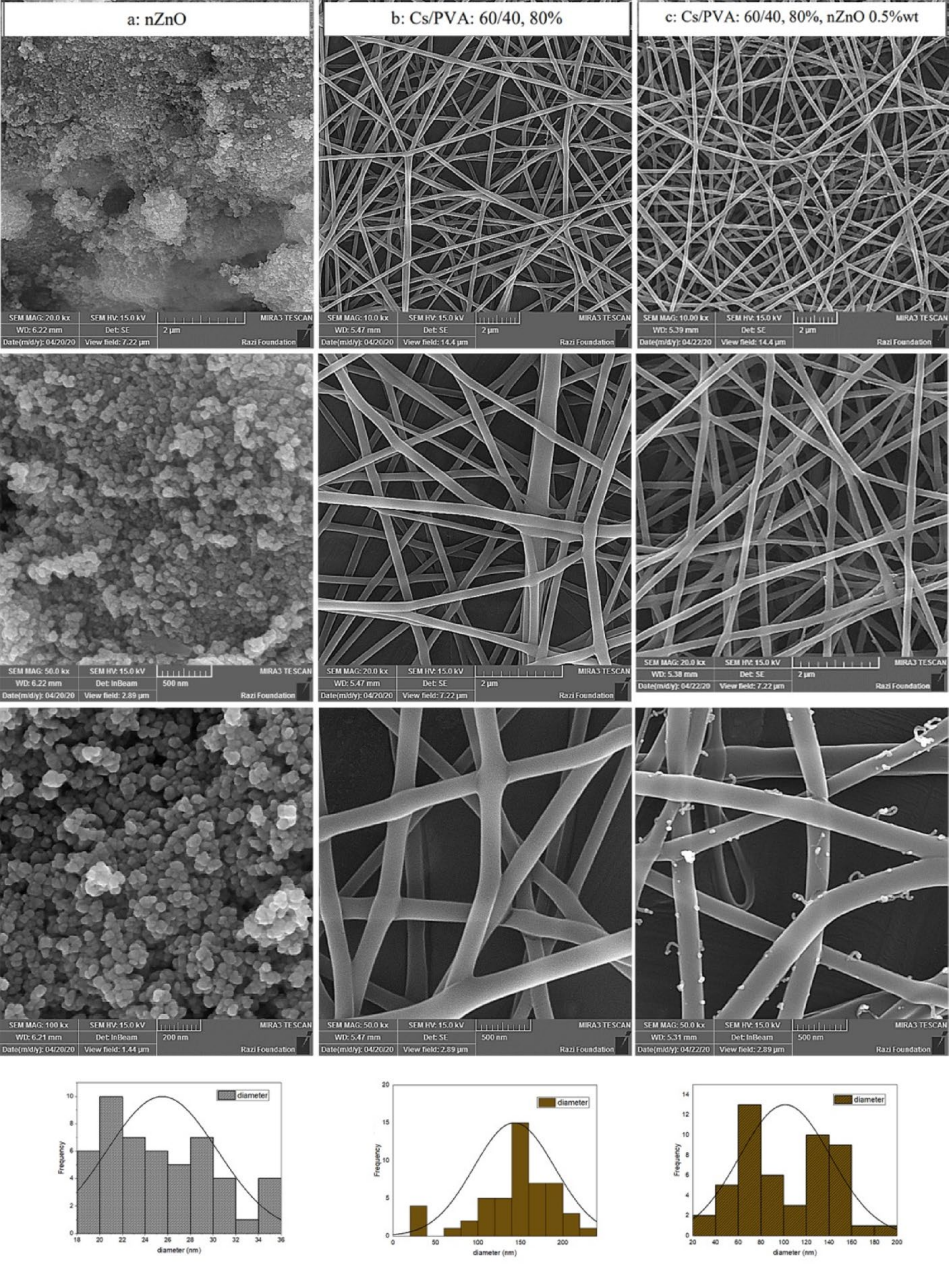
FE-SEM images and diameter distribution of the ZnO-NPs (column a), optimized treatment of Cs/PVA (column b), and composite Cs/PVA/ZnO-NPs (column c) in different magnifications (10x, 20x, 50x, and 100x).

Figure 4a depicts SEM images of the synthesized nanoparticles of ZnO and their diameter distribution at different magnifications. The nanoparticle formation of zinc oxide was spherical, and the diameter distribution was less than 50 nm. The behavior of nanoparticles was aggregating and in cluster form, according to the microscopic images. The synthesized nanoparticles were added to the optimized treatment (chitosan/PVA ratio: 60:40 with AcAcid 80%) by using uniaxial electrospinning. Figure 4b displays the smooth and continuous morphology of the nanofibers which were formed perfectly. The nanoparticles were scattered on the surface of the nanofibers with an obvious decrease in the nanofiber diameter compared to the unloading scaffold (Fig. 4c**)**. The nanoparticles dispersed on the surface of nanofibers confirmed a developed system with good compatibility between the polymers, nanoparticles, and solvent. The groups of –NH_3_^+^ in chitosan are generated from the –NH_2_ groups of AcAcid which have a definite effect on the ionic conductivity. Increasing the chitosan amount beside the nanoparticle can intensify this effect^65^.

The low viscosity of the composite solution embedded with ZnO-NPs increased the probability of bead formation. Also, the nanoparticles were the reason for the incontestable decrease in the average diameter of fibers through their effect on viscosity. The histograms of nanofiber diameters depict lower values in the Cs/PVA/ZnO-NPs composite compared to Cs/PVA. There is entanglement between the chains with more resistance for jet formation at higher viscosities, thus requiring more elevated voltages for the electrospinning. The addition of nanoparticles probably increases the conductivity of the solution, which facilitates the electrospinning process^11^.

**Fig. 5:**
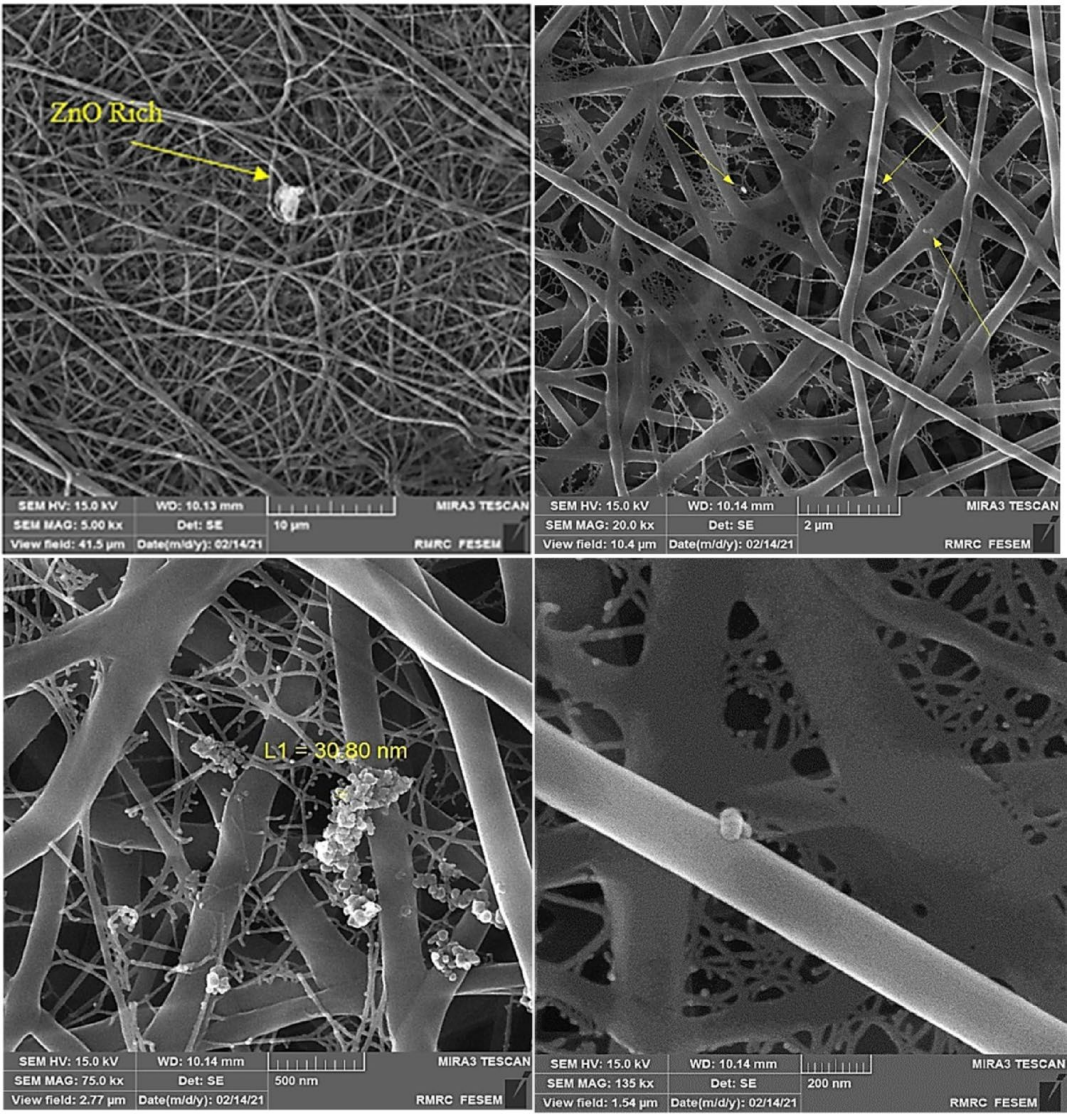
FE-SEM map images of nanocomposite Cs/PVA/ZnO-NPs in different magnifications.

**Fig. 6:**
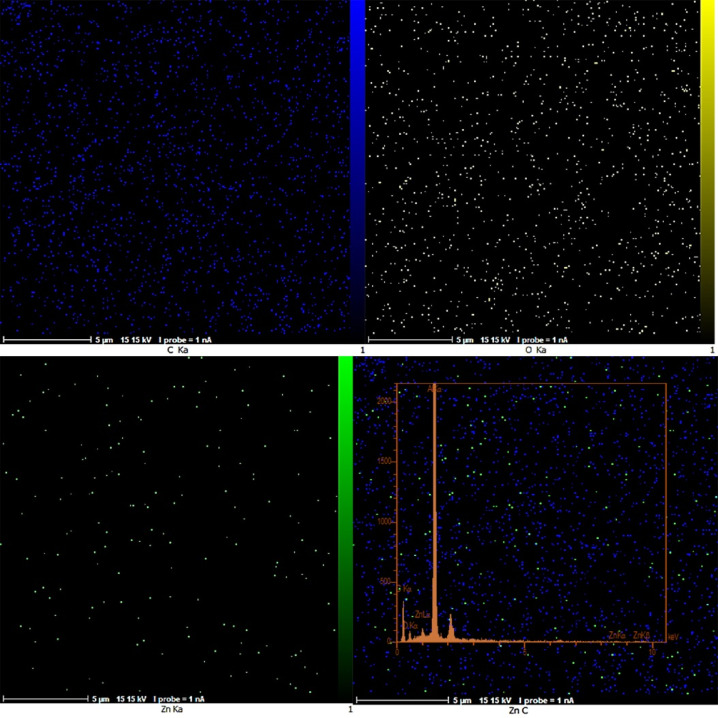
Elemental map distribution of Cs/PVA/ZnO-NPs composite: carbon (blue), oxygen (yellow), and Zn (green).

The elemental maps of fiber composites containing ZnO-NPs provided further information about the distribution of the nanoparticles according to X-ray energy scattering spectroscopy, a suitable method for quantitative and qualitative analysis of elemental composition on small scales. Figures 5 and 6 show the MAP analysis and EDS images of the nanocomposite samples Cs/PVA/ZnO-NPs which will demonstrate the presence of the main elements and the successful loading of nanoparticles on the fibers. Based on these maps, zinc was the characteristic element of ZnO (Green points), and Carbone (blue points) and Oxygen (yellow points) elements were detected. Also, based on this elemental map, ZnO-NPs (green points) were distributed homogeneously on the scaffold of Cs/PVA. Data analysis estimated the values of each component, carbon, oxygen, and zinc, as 56.09, 5.58, and 0.76%, respectively.

### FTIR and XRD

The functional groups of Cs, Cs/PVA, and Cs/PVA/ZnO-NPs were analyzed using FTIR spectroscopy. Figure 1 reveals the corresponding peaks of each polymer and their interactions. The spectra of Cs/PVA showed a broad band at 3100–3500 cm^− 1^, indicating –OH vibration stretching that could overlap with the –NH bands of amine and amide. The peaks at 1642 cm^− 1^ and 1577 cm^− 1^ are due to the interaction between the –OH and –NH bending vibrations of Cs /PVA, and the peak at 1085 cm^− 1^ could be related to the O–C vibration stretching group. The peak of 1148 cm^− 1^ indicates the formation of the acetal ring through cross-linking interaction. The apex between 1750 cm^− 1^ and 1735 cm^− 1^ is due to the C = O and O–C stretching of the acetate groups of PVA, and the peak at 1148 cm^− 1^ indicates the interaction between Cs and PVA^56^. PVA powder shows a sharp asymmetric carboxylate bond around 1635 cm^− 1^ and a hydroxyl bond close to 3444 cm^− 1^. The bands related to this polymer can be shifted to higher wavelengths with different intensities after mixing with the second polymer^30^. Yang et al. (2018)^63^ stated that the slight broadening of the hydroxyl peak observed in the band of 3444 cm^− 1^ could be due to some intramolecular hydrogen bond that occurred during solution preparation. Also, changing the position of the carboxylate band to a higher wave number can indicate the interaction between the polymers that occurred through hydrogen bonds between the etheric oxygen of the PVA and the hydroxyl group of the chitosan.

According to previous studies, the Cs/PVA composite exhibits H–C vibration around 2937 cm^− 1^ shifts to slightly lower wavelengths in pure chitosan. The peaks around 1652 cm^− 1^ and 1590 cm^− 1^ are related to the alternation of amide I and amide II groups, which slightly changes to the wavelength of 1565 cm^− 1^ in the Cs/PVA composite. The peak at 1370 cm^− 1^ corresponds to C–OH stretching vibration, which increased with increasing PVA. In addition, characteristic bonds for saccharide structures can be observed at 895 cm^− 1^, 1060 cm^− 1^, and 1152 cm^− 1^, which do not show apparent changes in the combined system of functional groups^63^. In general, metal oxides show absorption bonds in the region below 1000 cm^− 1^, and the characteristic peak of ZnO appears in the range of 500–700 cm^− 1^. The peak at around 453 cm^− 1^ and 800 cm^− 1^ can be corresponding to the Zn-O stretch and vibration mode of Zn-O-Zn^62,67^. In addition, the absorption peaks at 1596 cm^− 1^, 1404 cm^− 1^, and 453 cm^− 1^ in the composite could be because of the ZnO nanoparticles presence. The peaks between 1750 and 520 cm^− 1^ correspond to the vibration stretching of ZnO, a clear shift indicating that the ZnO particles are integrated with the engaged polymers completely^50^. The characteristic peak at 1560 cm^− 1^ could be due to the vibrational stretching of the Oxygen-Zn bond. Also, various amounts of zinc oxide in the composite caused broader peaks instead of the sharp peaks of pure zinc oxide^30^. Figure 1 shows the XRD patterns of Cs, Cs/PVA, and Cs/PVA/ZnO-NPs. The appearance of new peaks above 20° specified the presence of PVA and oxidized nanoparticles. Also, the zenith at around 10° and 20° in Cs/PVA nanofibers indicates the strong interaction between the polymers and reducing the chitosan crystallinity. X-ray diffraction results provide evidence for FTIR spectroscopy results showing that some interactions occurred between chitosan and PVA^4^. Gutha et al. (2017)^50^ reported that pure chitosan showed peaks at 2^θ^ of 10° and 20° and the composite with lower density at 2^θ^ of 19.70°, and 11.50°. Also, according to the report of Yang et al. (2018)^63^, pure chitosan showed a diffraction peak of 20.20° that disappeared in the Cs/PVA composite because of the good compatibility between the polymers. In PVA, two peaks around 16.00° and 22.50° broadened in the Cs/PVA composite, which indicates a decrease in the initial crystallinity of PVA. If the two polymers are not well mixed, all the peaks of the two polymers should be observed separately in the XRD pattern. Any weak interaction between the mixtures of polymers can cause amorphous behavior^30^. Also, it could be confirmed that the electrospinning process has reduced the crystallization of the polymer structure. In the study of Shalumon et al. (2011)^30^, the pattern of zinc oxide nanoparticles shows its diffraction peaks at 2^θ^ of 20.33° and 30.36°, and in the sodium-alginate/PVA/ZnO composite, specific peaks of nanoparticles appeared at 17.20°, 38.60°, 44.80°, 65°, and 78.20°. Also, the concentration increase of nanoparticles in the mixture increased the intensity of the peaks. The new zenith of the diffraction pattern can be due to the formation of new covalent bonds between nanoparticles and composite fibers. The increase in intensity may be due to the new bonds of zinc and oxygen molecules.

### Antibacterial assay

**Fig. 7:**
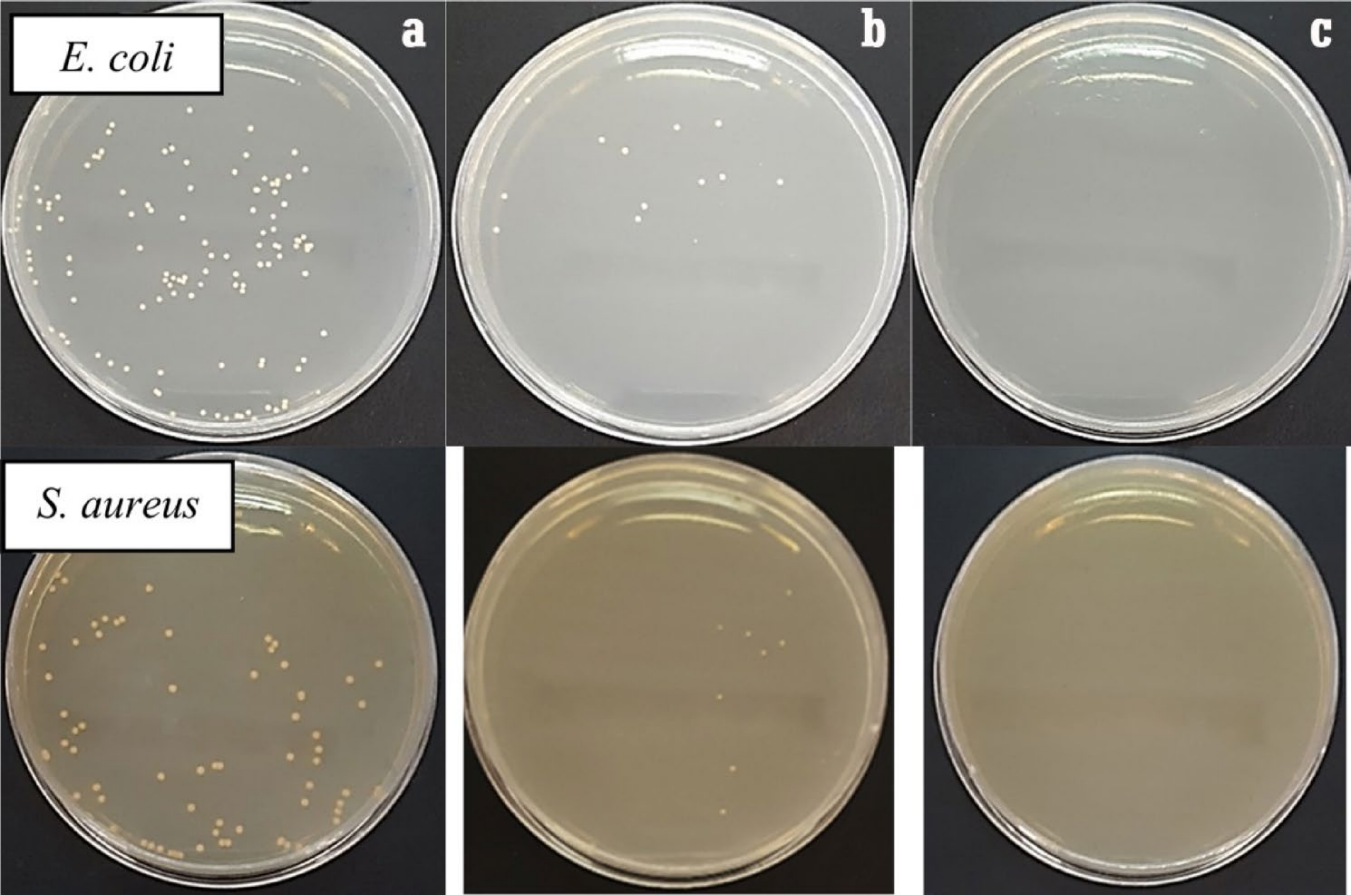
Antibacterial activity and comparison of colony growth inhibitions, control (**a**), Cs/PVA (**b**), and Cs/PVA/ZnO-NPs.

The antibacterial activity of the Cs/PVA and Cs/PVA/ZnO-NPs composites were evaluated according to colony survival counting (Fig. 7). Based on the results, the antibacterial activities of Cs/PVA and Cs/PVA/ZnO-NPs against *E. coli* were 85.33% and 99.33%, respectively. The antibacterial activity of scaffolds against *S. aureus* also showed a high inhibitory capacity of 90.00% and 99.93%, respectively, which destroyed the colony’s population. This feature of the polymer can be related to several functions, such as the polycationic nature of the polymer, which interacts with anionic groups on the surface to alter membrane permeability, performance, and cell structure. Abdelgawad et al. (2014)^65^ reported that in different ratios of PVA/Cs (10:90, 20:80, and 40:60), a relative antibacterial activity was observed with an increase in the ratio of chitosan. It was stated that the polymer concentration facilitates the mobility and formation of hydrogen and covalent bonds between the functional groups of the chitosan chains. Also, the results of Goy et al. (2016)^68^ showed that chitosan and its derivatives had convincing effects on the growth log reduction of *S. aureus* and *E. coli* bacteria. The peptidoglycan layer in gram-positive bacteria is a serious issue for the adequate performance of antibiotic substances because it effectively develops a high resistance against the death of microorganisms, which also indicates severe antibacterial activity of the extracted polymer and the composite. The positive charge of the amine groups of chitosan chains generates an electrostatic interaction with anionic groups on the bacterial cell membrane that raptures the cell integrity by changing the permeability structures^63,69^.

Furthermore, chitosan can chelate the vital elements of the cell and disrupt the enzymatic reactions of microorganisms^33^. The initial release of zinc oxide nanoparticles from the scaffolds is another aspect of the inhibition behavior of the composite. According to the reports, the inhibition rate depends on the sufficient concentration of chitosan and nanoparticles in the structure of the dressing. Sarhan and Azzazy (2015)^62^ stated that increasing the incubation time of exposure could improve the antibacterial activity according to the degradation process which ultimately increases the concentration of antibacterial substances in the wound. At higher bacterial concentrations, only the treatment with a higher percentage of chitosan was able to inhibit colonial growth^65^. Our results confirm the study on the investigation of the antibacterial activity of chitosan/polyvinyl alcohol/honey against *S. aureus* bacteria. The results of Sarhan and Azzazy (2015)^62^ showed that an increase in the chitosan concentration in the composite improved the antibacterial activity. Among the factors, increasing the incubation time, concentration, and combination of polymers have a definite role in the antibacterial activity. Furthermore, the structure of the nanofiber network does not increase the bacterial activity of the composite and certain functionality comes from the high rates of surface-to-volume of the nanofibers which stimulate the antibacterial functionality. The nanoparticles placed on the nanofibers can converted into an encapsulated state, allowing their release from the nanofibers and diffusion into the culture medium, which effectively restricts bacterial growth^62^.

### Cytotoxicity evaluation

**Fig. 8:**
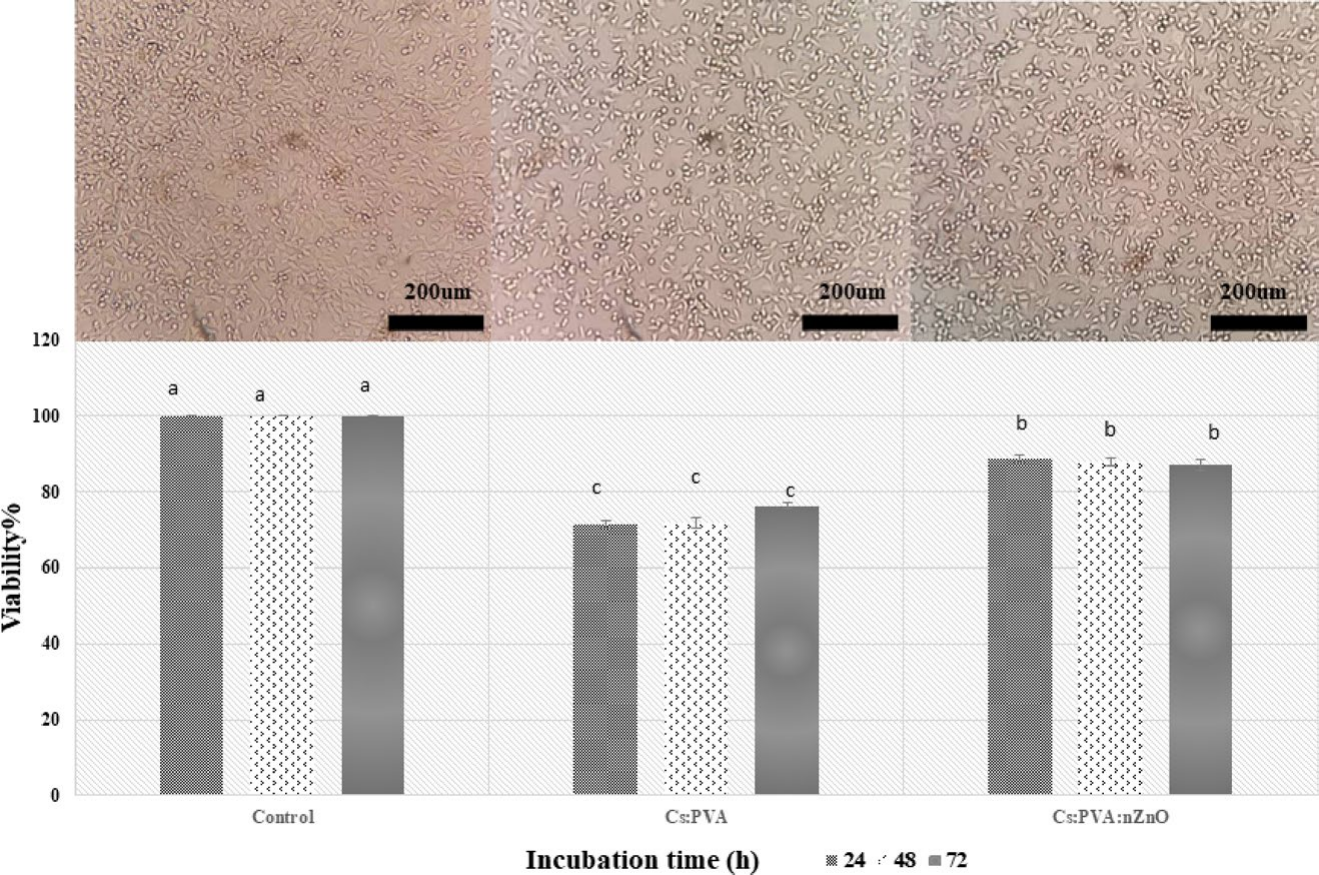
Cytotoxicity evaluation and inverted microscopic images of L9L29 mouse fibroblast cells for up to72 hours of incubation: control, Cs/PVA, and Cs/PVA/NPs. Data are the mean ± SD of *n* = 3. Different letters indicate a significant difference, p< (0.05).

The proliferative capacity of mouse fibroblasts on electrospun scaffolds was evaluated through an MTT assay. Mouse primary dermal fibroblasts were cultured directly on the Cs/PVA and Cs/PVA/ZnO-NPs composite and negative control for 72 h (Fig. 8). In the toxicology experiment, the extraction medium of the prepared scaffolds had no toxic effect on the fibroblast cells’ survival. The cell viability of the extraction medium was more than 86.99% in Cs/PVA/ZnO-NPs which confirmed the non-toxicity of the chitosan and the composite. The cytotoxicity results showed a significant difference in cell viability among the various treatments. Although the viability rate was high across all treatments, the Cs/PVA/ZnO-NPs treatment exhibited higher viability compared to Cs/PVA, demonstrating excellent cell viability among the treatments (*p* < 0.05). However, no significant difference was observed between incubation times within each treatment. The optical concentration of mouse skin fibroblast cells in the samples gradually increased which indicates the metabolic consistency of the fibroblast cells on cultured scaffolds^62^. This may be due to the fact that low concentrations of ZnO-NPs can enhance fibroblast proliferation by influencing the expression of proteins associated with cell growth. In contrast, higher concentrations induce oxidative stress and cytotoxicity^70^. Therefore, from the outset, the focus was on using low concentrations of nanoparticles. The chitosan has improved the hydrophilicity of the scaffold and provided better cellular interaction which was also confirmed by the contact angle. Sarhan and Azzazy (2015)^62^ reported the cells cultured with chitosan showed no significant difference in cell viability compared to negative control. It was also stated a noteworthy discrepancy in improved survival compared to positive control. An investigation on the cytotoxicity of alginate/PVA nanofibers containing 0.5 and 1% ZnO nanoparticles on the fibroblast cells showed a slight decrease in optical density after 48 h of incubation, while the cell proliferation increased after 98 h, compared to the control. They reported at the concentrations of 0.5% ZnO, the consistency and development are acceptable compared to higher concentrations of nanoparticles. It is necessary to determine the optimal concentration of zinc oxide with the lowest toxicity and good antibacterial activity^30^.

### Contact angle and mechanical behavior

**Fig. 9:**
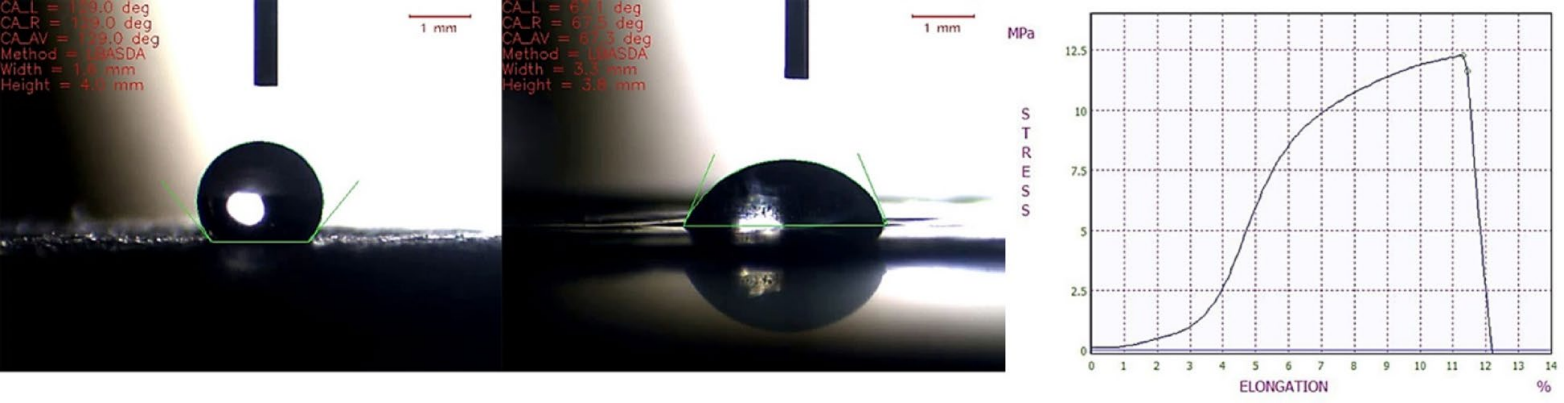
Water contact angle of Cs and Cs/PVA/ZnO-NPs and tensile strength of Cs/PVA/ZnO-NPs.

The contact angle exhibits the hydrophilicity of Cs and Cs/PVA/ZnO-NPs composite (Fig. 9) that was 129^o^ and 67.30^o^, respectively. Usually, the contact angle of hydrophilic surfaces is lower than that of hydrophobic ones. According to the study of Pinto et al. (2018)^71^, the low interaction of the chitosan biofilm with water shows the hydrophobic nature of chitosan. Also, according to the results, the addition of nanoparticles increased the hydrophilicity of the composite compared to the original polymer. This can be due to the hydrogen bonds of ZnO nanoparticles which occurred with water molecules. Rotta et al. (2009)^72^ stated that the addition of the second polymer to chitosan decreased its contact angle from 95^o^ to 70^o^ and reduced its hydrophobicity.

This feature of chitosan can determine its potential for wound dressings because of the interaction that occurs between the damaged surface and the composite dress. Hydrophilicity or hydrophobicity of the scaffolds can play a very critical role in the end product. Improving the hydrophilicity of the cover can facilitate the proliferation and cell adhesion process. Changing the ratio of each polymer’s participation can control the solubility or insolubility of the composite that has the integrity of the target application as a wound covering, implant, or packaging. In the current study, the composite revealed a contact angle of less than 90^o^ with good hydrophilicity. El-Hefian et al. (2010)^73^ stated that chitosan and PVA are hydrophilic materials that are soluble in acidic and aqueous environments. A general decrease in water contact angle occurs with the increase in PVA. The contact angle of the composite showed a value less ​​than the pure chitosan. Improving the hydrophilicity of the nano-fibrous surface can enhance cell survival and interactions. Not enough surfaces for cellular interactions can decrease the cell’s movements for potential use in medical applications.

The tensile test investigated the mechanical properties of the composite nanofiber at room temperature. The composite must reveal appropriate elongation at break for wound covering purposes to dress the wound without rupturing. Figure 9 also shows the tensile stress-strain curves for composite nanofibers with a thickness of 0.02 mm. The tensile strength of electrospun nanofibers was between 12.26 and 11.63 MPa at peak and breakpoints, respectively, which is good for wound dressing application. The addition of PVA along with Cs can affect the flexibility of the composite with only minor changes in tensile strength^74^. El-Hefian et al. (2010)^69^ worked on the tensile strength and elongation at the breaking point of chitosan/PVA composite films. Their results showed that the combination of polymers improved the tensile strength and decreased the elongation, rather than each other by itself. This improvement in tensile strength can be due to the interaction between the hydroxyl and amino groups of chitosan and PVA. The amount of tensile strength can also show the proper relation and chemical bonds accrued between the polymers^61^. The combination of ZnO-NPs with CS/PVA results in the formation of hydrogen bonds between the functional groups of chitosan (such as amino groups) and PVA (such as hydroxyl groups) with the surface of ZnO-NPs, which creates a stronger network and restricts the movement of polymer chains, which in turn increases tensile strength. Also, these bonds create a more uniform distribution of ZnO-NPs in the polymer matrix, preventing their aggregation, which in turn helps improve mechanical properties^75,76^ .

## Conclusion

In the current study, the Cs extraction was optimized to reduction of chemical using and time of extraction. Extracted Cs was used for nanofiber production along with nanoparticle loading in a continuous procedure. The Cs and PVA mixture was successfully electrospun and a good combination of polymers, solvent, and the ratios developed through an optimization process (10wt. % PVA and 1wt. % CS in AcAcetic 80%). Afterward, the ZnO-NPs were incorporated into the nanofiber scaffold, and their structural, physicomechanical, antibacterial activity, and in vitro properties were evaluated. The FE-SEM analysis proved the smooth and bead-free morphology of the fibers. The elemental analysis also revealed a good distribution of nanoparticles in the scaffold. The ZnO-NPs improved the hydrophilicity and antibacterial activity of the scaffold for application in wound covering. The results showed the nontoxicity of CS/PVA/ZnO-NPs composite and its considerable potential for future application in wound dressing.

## Data Availability

The data supporting the findings of this study are available through corresponding author upon reasonable request.

## References

[1] Hamdi, M. et al. Chitin extraction from blue crab (Portunus segnis) and shrimp (Penaeus kerathurus) shells using digestive alkaline proteases from P. segnis viscera. *Int. J. Biol. Macromol.* **101**, 455–463. 10.1016/j.ijbiomac.2017.02.103 (2017). 28336276 10.1016/j.ijbiomac.2017.02.103

[2] Mawazi, S. M., Kumar, M., Ahmad, N., Ge, Y. & Mahmood, S. Recent applications of chitosan and its derivatives in antibacterial, anticancer, wound healing, and tissue engineering fields. *Polym. (Basel)*. 16. 10.3390/polym16101351 (2024). 10.3390/polym16101351 PMC11125164 38794545

[3] Yousefi, M. et al. Nanofabrication of chitosan-based dressing to treat the infected wounds: in vitro and in vivo evaluations. *Future Sci. OA*. **10** (Fso921). 10.2144/fsoa-2023-0077 (2024). 10.2144/fsoa-2023-0077 PMC11140651 38827799

[4] Habiba, U. et al. Effect of deacetylation on property of electrospun Chitosan/PVA nanofibrous membrane and removal of Methyl orange, Fe(III) and Cr(VI) ions. *Carbohydr. Polym.* **177**, 32–39. 10.1016/j.carbpol.2017.08.115 (2017). 28962774 10.1016/j.carbpol.2017.08.115

[5] Matei, A., Stoian, M., Brincoveanu, O. & Ţucureanu, V. Preparation and characterization of nanocomposites based on Chitosan with ZnO-Curcumin. *Ceram. Int.* **49**, 19829–19839. 10.1016/j.ceramint.2023.03.100 (2023).

[6] El Knidri, H., El Khalfaouy, R., Laajeb, A., Addaou, A. & Lahsini, A. Eco-friendly extraction and characterization of chitin and chitosan from the shrimp shell waste via microwave irradiation. *Process Saf. Environ. Prot.* **104**, 395–405. 10.1016/j.psep.2016.09.020 (2016).

[7] Sedaghat, F., Yousefzadi, M., Toiserkani, H. & Najafipour, S. Bioconversion of shrimp waste Penaeus merguiensis using lactic acid fermentation: an alternative procedure for chemical extraction of chitin and chitosan. *Int. J. Biol. Macromol.* **104**, 883–888. 10.1016/j.ijbiomac.2017.06.099 (2017). 28663153 10.1016/j.ijbiomac.2017.06.099

[8] Geng, X., Kwon, O. H. & Jang, J. Electrospinning of chitosan dissolved in concentrated acetic acid solution. *Biomaterials* **26**, 5427–5432. 10.1016/j.biomaterials.2005.01.066 (2005). 15860199 10.1016/j.biomaterials.2005.01.066

[9] Torres-Giner, S., Ocio, M. J. & Lagaron, J. M. Development of active antimicrobial fiber-based chitosan polysaccharide nanostructures using electrospinning. *Eng. Life Sci.* **8**, 303–314. 10.1002/elsc.200700066 (2008).

[10] Homayoni, H., Ravandi, S. A. H. & Valizadeh, M. Influence of the molecular weight of chitosan on the spinnability of chitosan/poly(vinyl alcohol) blend nanofibers. *J. Appl. Polym. Sci.* **113**, 2507–2513. 10.1002/app.30148 (2009).

[11] Shalumon, K. T. et al. Single step electrospinning of chitosan/poly(caprolactone) nanofibers using formic acid/acetone solvent mixture. *Carbohydr. Polym.* **80**, 413–419. 10.1016/j.carbpol.2009.11.039 (2010).

[12] Sudheesh Kumar, P. T. et al. Flexible and microporous chitosan hydrogel/nano ZnO composite bandages for wound dressing: in vitro and in vivo evaluation. *ACS Appl. Mater. Interfaces*. **4**, 2618–2629. 10.1021/am300292v (2012). 22489770 10.1021/am300292v

[13] Kohsari, I., Shariatinia, Z. & Pourmortazavi, S. M. Antibacterial electrospun chitosan-polyethylene oxide nanocomposite Mats containing ZIF-8 nanoparticles. *Int. J. Biol. Macromol.* **91**, 778–788. 10.1016/j.ijbiomac.2016.06.039 (2016). 27311504 10.1016/j.ijbiomac.2016.06.039

[14] Hu, X. et al. Electrospinning of polymeric nanofibers for drug delivery applications. *J. Control Release*. **185**, 12–21. 10.1016/j.jconrel.2014.04.018 (2014). 24768792 10.1016/j.jconrel.2014.04.018

[15] Duan, B., Dong, C., Yuan, X. & Yao, K. Electrospinning of Chitosan solutions in acetic acid with Poly (ethylene oxide). *J. Biomater. Sci. Polym. Ed.* **15**, 797–811 (2004). 15255527 10.1163/156856204774196171

[16] Scaffaro, R. & Lopresti, F. Properties-morphology relationships in electrospun Mats based on polylactic acid and graphene nanoplatelets. *Compos. Part A: Appl. Sci. Manufac.* **108**, 23–29 (2018).

[17] Cipitria, A., Skelton, A., Dargaville, T., Dalton, P. & Hutmacher, D. Design, fabrication and characterization of PCL electrospun scaffolds—a review. *J. Mater. Chem.* **21**, 9419–9453 (2011).

[18] Chong, W. J. et al. Biodegradable PLA-ZnO nanocomposite biomaterials with antibacterial properties, tissue engineering viability, and enhanced biocompatibility. *Smart Mater. Manuf.* **1**, 100004. 10.1016/j.smmf.2022.100004 (2023).

[19] Karthikeyan, C., Varaprasad, K., Akbari-Fakhrabadi, A., Hameed, A. S. H. & Sadiku, R. Biomolecule Chitosan, Curcumin and ZnO-based antibacterial nanomaterial, via a one-pot process. *Carbohydr. Polym.* **249**, 116825. 10.1016/j.carbpol.2020.116825 (2020). 32933672 10.1016/j.carbpol.2020.116825

[20] Pino, P., Bosco, F., Mollea, C. & Onida, B. Antimicrobial Nano-Zinc oxide biocomposites for wound healing applications: A review. *Pharmaceutics* **15**10.3390/pharmaceutics15030970 (2023). 10.3390/pharmaceutics15030970 PMC10053511 36986831

[21] Liu, Y. et al. Antibacterial activities of zinc oxide nanoparticles against Escherichia coli O157:H7. *J. Appl. Microbiol.* **107**, 1193–1201. 10.1111/j.1365-2672.2009.04303.x (2009). 19486396 10.1111/j.1365-2672.2009.04303.x

[22] Delavari, M. M. & Stiharu, I. Preparing and characterizing novel biodegradable Starch/PVA-based films with nano-sized Zinc-Oxide particles for wound-dressing applications. *Appl. Sci.* **12**, 4001 (2022).

[23] Nasiri, G. et al. Fabrication and evaluation of Poly (vinyl alcohol)/gelatin fibrous scaffold containing ZnO nanoparticles for skin tissue engineering applications. *Mater. Today Commun.* **33**, 104476. 10.1016/j.mtcomm.2022.104476 (2022).

[24] Khorasani, M. T., Joorabloo, A., Adeli, H., Milan, P. B. & Amoupour, M. Enhanced antimicrobial and full-thickness wound healing efficiency of hydrogels loaded with heparinized ZnO nanoparticles: in vitro and in vivo evaluation. *Int. J. Biol. Macromol.* **166**, 200–212. 10.1016/j.ijbiomac.2020.10.142 (2021). 33190822 10.1016/j.ijbiomac.2020.10.142

[25] Bagheri, M., Validi, M., Gholipour, A., Makvandi, P. & Sharifi, E. Chitosan nanofiber biocomposites for potential wound healing applications: antioxidant activity with synergic antibacterial effect. *Bioeng. Transl Med.* **7**, e10254. 10.1002/btm2.10254 (2022). 35111951 10.1002/btm2.10254 PMC8780905

[26] Benhabiles, M. S. et al. Antibacterial activity of Chitin, Chitosan and its oligomers prepared from shrimp shell waste. *Food Hydrocoll.* **29**, 48–56. 10.1016/j.foodhyd.2012.02.013 (2012).

[27] Charoenvuttitham, P., Shi, J. & Mittal, G. S. Chitin extraction from black tiger shrimp (Penaeus monodon) waste using organic acids. *Sep. Sci. Technol.* **41**, 1135–1153. 10.1080/01496390600633725 (2006).

[28] Tolaimate, A., Desbrieres, J., Rhazi, M. & Alagui, A. Contribution to the Preparation of chitins and chitosans with controlled physico-chemical properties. *Polymer* **44**, 7939–7952. 10.1016/j.polymer.2003.10.025 (2003).

[29] Kurita, K. et al. Squid chitin as a potential alternative chitin source: deacetylation behavior and characteristic properties. *J. Polym. Sci., Part A: Polym. Chem.* **31**, 485–491. 10.1002/pola.1993.080310220 (1993).

[30] Shalumon, K. T. et al. Sodium alginate/poly(vinyl alcohol)/nano ZnO composite nanofibers for antibacterial wound dressings. *Int. J. Biol. Macromol.* **49**, 247–254. 10.1016/j.ijbiomac.2011.04.005 (2011). 21635916 10.1016/j.ijbiomac.2011.04.005

[31] AOAC. Official methods of analysis. Maryland, USA. (1990).

[32] Rinaudo, M. Chitin and Chitosan: properties and applications. *Prog. Polym. Sci.* **31**, 603–632. 10.1016/j.progpolymsci.2006.06.001 (2006).

[33] Kumari, S., Kumar Annamareddy, S. H., Abanti, S. & Kumar Rath, P. Physicochemical properties and characterization of Chitosan synthesized from fish scales, crab and shrimp shells. *Int. J. Biol. Macromol.* **104**, 1697–1705. 10.1016/j.ijbiomac.2017.04.119 (2017). 28472681 10.1016/j.ijbiomac.2017.04.119

[34] Hajji, S. et al. Structural differences between Chitin and Chitosan extracted from three different marine sources. *Int. J. Biol. Macromol.* **65**, 298–306. 10.1016/j.ijbiomac.2014.01.045 (2014). 24468048 10.1016/j.ijbiomac.2014.01.045

[35] Abdelgawad, A. M., Hudson, S. M. & Rojas, O. J. Antimicrobial wound dressing nanofiber Mats from multicomponent (chitosan/silver-NPs/polyvinyl alcohol) systems. *Carbohydr. Polym.* **100**, 166–178. 10.1016/j.carbpol.2012.12.043 (2014). 24188851 10.1016/j.carbpol.2012.12.043

[36] Younes, I. et al. Optimization of proteins and minerals removal from shrimp shells to produce highly acetylated chitin. *Int. J. Biol. Macromol.* **84**, 246–253. 10.1016/j.ijbiomac.2015.08.034 (2016). 26299708 10.1016/j.ijbiomac.2015.08.034

[37] Ghorbel-Bellaaj, O. et al. Shrimp waste fermentation with Pseudomonas aeruginosa A2: optimization of chitin extraction conditions through Plackett-Burman and response surface methodology approaches. *Int. J. Biol. Macromol.* **48**, 596–602. 10.1016/j.ijbiomac.2011.01.024 (2011). 21300086 10.1016/j.ijbiomac.2011.01.024

[38] Younes, I. et al. Chitin extraction from shrimp shell using enzymatic treatment. antitumor, antioxidant and antimicrobial activities of chitosan. *Int. J. Biol. Macromol.* **69**, 489–498. 10.1016/j.ijbiomac.2014.06.013 (2014). 24950313 10.1016/j.ijbiomac.2014.06.013

[39] Mohanasrinivasan, V. et al. Studies on heavy metal removal efficiency and antibacterial activity of chitosan prepared from shrimp shell waste. *3 Biotech.* **4**, 167–175. 10.1007/s13205-013-0140-6 (2014). 28324448 10.1007/s13205-013-0140-6 PMC3964254

[40] Apriyanti, D. T., Susanto, H. & Rokhati, N. Influence of microwave irradiation on extraction of chitosan from shrimp shell waste. 6, (2018). 10.14710/reaktor.18.1.45-50 (2018).

[41] Cheng, J. et al. The physicochemical properties of chitosan prepared by microwave heating. *Food Sci. Nutr.* **8**, 1987–1994. 10.1002/fsn3.1486 (2020). 32328265 10.1002/fsn3.1486 PMC7174223

[42] El Knidri, H., Dahmani, J., Addaou, A., Laajeb, A. & Lahsini, A. Rapid and efficient extraction of chitin and chitosan for scale-up production: effect of process parameters on deacetylation degree and molecular weight. *Int. J. Biol. Macromol.* **139**, 1092–1102. 10.1016/j.ijbiomac.2019.08.079 (2019). 31404606 10.1016/j.ijbiomac.2019.08.079

[43] Mahdy Samar, M., El-Kalyoubi, M. H., Khalaf, M. M. & Abd El-Razik, M. M. Physicochemical, functional, antioxidant and antibacterial properties of chitosan extracted from shrimp wastes by microwave technique. *Annals Agricultural Sci.* **58**, 33–41. 10.1016/j.aoas.2013.01.006 (2013).

[44] Abd El-Hack, M. E. et al. Antimicrobial and antioxidant properties of chitosan and its derivatives and their applications: A review. *Int. J. Biol. Macromol.* **164**, 2726–2744. 10.1016/j.ijbiomac.2020.08.153 (2020). 32841671 10.1016/j.ijbiomac.2020.08.153

[45] Bełdowski, P. et al. Effect of Chitosan deacetylation on its affinity to type III collagen: A molecular dynamics study. *Mater. (Basel)*. **15**10.3390/ma15020463 (2022). 10.3390/ma15020463 PMC8781747 35057179

[46] Younes, I. & Rinaudo, M. Chitin and chitosan preparation from marine sources. structure, properties and applications. *Mar. Drugs*. **13**, 1133–1174 (2015). 25738328 10.3390/md13031133 PMC4377977

[47] Kumar, M. R., Muzzarelli, R. A., Muzzarelli, C., Sashiwa, H. & Domb, A. Chitosan chemistry and pharmaceutical perspectives. *Chem. Rev.* **104**, 6017–6084 (2004). 15584695 10.1021/cr030441b

[48] Austin, P. R., Brine, C. J., Castle, J. E., Zikakis, J. P. & Chitin New facets of research. *Science* **212**, 749. 10.1126/science.7221561 (1981). 7221561 10.1126/science.7221561

[49] Abdou, E. S., Nagy, K. S. A. & Elsabee, M. Z. Extraction and characterization of Chitin and Chitosan from local sources. *Bioresour. Technol.* **99**, 1359–1367. 10.1016/j.biortech.2007.01.051 (2008). 17383869 10.1016/j.biortech.2007.01.051

[50] Gutha, Y., Pathak, J. L., Zhang, W., Zhang, Y. & Jiao, X. Antibacterial and wound healing properties of chitosan/poly(vinyl alcohol)/zinc oxide beads (CS/PVA/ZnO). *Int. J. Biol. Macromol.* **103**, 234–241. 10.1016/j.ijbiomac.2017.05.020 (2017). 28499948 10.1016/j.ijbiomac.2017.05.020

[51] Ghazalian, M., Afshar, S., Rostami, A., Rashedi, S. & Bahrami, S. H. Fabrication and characterization of chitosan-polycaprolactone core-shell nanofibers containing Tetracycline hydrochloride. *Colloids Surf., A*. **636**, 128163. 10.1016/j.colsurfa.2021.128163 (2022).

[52] Teli, M. D. & Sheikh, J. Extraction of Chitosan from shrimp shells waste and application in antibacterial finishing of bamboo Rayon. *Int. J. Biol. Macromol.* **50**, 1195–1200. 10.1016/j.ijbiomac.2012.04.003 (2012). 22522048 10.1016/j.ijbiomac.2012.04.003

[53] Nouri, M., Khodaiyan, F., Razavi, S. H. & Mousavi, M. Improvement of Chitosan production from Persian Gulf shrimp waste by response surface methodology. *Food Hydrocoll.* **59**, 50–58. 10.1016/j.foodhyd.2015.08.027 (2016).

[54] Srinivasan, H., Kanayairam, V. & Ravichandran, R. Chitin and Chitosan Preparation from shrimp shells Penaeus monodon and its human ovarian cancer cell line, PA-1. *Int. J. Biol. Macromol.* **107**, 662–667. 10.1016/j.ijbiomac.2017.09.035 (2018). 28923565 10.1016/j.ijbiomac.2017.09.035

[55] Kucukgulmez, A. et al. Physicochemical characterization of Chitosan extracted from Metapenaeus stebbingi shells. *Food Chem.* **126**, 1144–1148. 10.1016/j.foodchem.2010.11.148 (2011).

[56] De Antonino, Q. Preparation and characterization of chitosan obtained from shells of shrimp (Litopenaeus vannamei Boone). *Mar. Drugs*. **15**10.3390/md15050141 (2017). 10.3390/md15050141 PMC5450547 28505132

[57] Wanjun, T., Cunxin, W. & Donghua, C. Kinetic studies on the pyrolysis of chitin and chitosan. *Polym. Degrad. Stab.* **87**, 389–394. 10.1016/j.polymdegradstab.2004.08.006 (2005).

[58] Ou, C. et al. Effect of transition metal ions on the thermal degradation of chitosan. *Cogent Chem.* **2**, 1216247. 10.1080/23312009.2016.1216247 (2016).

[59] Lertwattanaseri, T., Ichikawa, N., Mizoguchi, T., Tanaka, Y. & Chirachanchai, S. Microwave technique for efficient deacetylation of chitin nanowhiskers to a Chitosan nanoscaffold. *Carbohydr. Res.* **344**, 331–335. 10.1016/j.carres.2008.10.018 (2009). 19111284 10.1016/j.carres.2008.10.018

[60] Kriegel, C., Kit, K. M., McClements, D. J. & Weiss, J. Electrospinning of chitosan–poly(ethylene oxide) blend nanofibers in the presence of micellar surfactant solutions. *Polymer* **50**, 189–200. 10.1016/j.polymer.2008.09.041 (2009).

[61] Gholipour-Kanani, A. et al. Tissue engineered poly(caprolactone)-chitosan-poly(vinyl alcohol) nanofibrous scaffolds for burn and cutting wound healing. *IET Nanobiotechnol.* **8**, 123–131. 10.1049/iet-nbt.2012.0050 (2014). 25014084 10.1049/iet-nbt.2012.0050

[62] Sarhan, W. A. & Azzazy, H. M. High concentration honey Chitosan electrospun nanofibers: biocompatibility and antibacterial effects. *Carbohydr. Polym.* **122**, 135–143. 10.1016/j.carbpol.2014.12.051 (2015). 25817652 10.1016/j.carbpol.2014.12.051

[63] Yang, S., Lei, P., Shan, Y. & Zhang, D. Preparation and characterization of antibacterial electrospun Chitosan/poly (vinyl alcohol)/graphene oxide composite nanofibrous membrane. *Appl. Surf. Sci.* **435**, 832–840. 10.1016/j.apsusc.2017.11.191 (2018).

[64] Ohkawa, K., Cha, D., Kim, H., Nishida, A. & Yamamoto, H. Electrospinning of chitosan. *Macromol. Rapid Commun.* **25**, 1600–1605. 10.1002/marc.200400253 (2004).

[65] Abdelgawad, A. M., Hudson, S. M. & Rojas, O. J. Antimicrobial wound dressing nanofiber Mats from multicomponent (chitosan/silver-NPs/polyvinyl alcohol) systems. *Carbohydr. Polym.* **100**, 166–178. 10.1016/j.carbpol.2012.12.043 (2014). 24188851 10.1016/j.carbpol.2012.12.043

[66] Hadjianfar, M., Semnani, D. & Varshosaz, J. An investigation on polycaprolactone/chitosan/Fe3O4 nanofibrous composite used for hyperthermia. *Polym. Adv. Technol.* **30**, 2729–2741. 10.1002/pat.4704 (2019).

[67] Babaei, A., Haji Abdolrasouli, M. & Rostami, A. Polylactic acid/polycaprolactone bionanocomposites containing zinc oxide nanoparticles: structure, characterization and cytotoxicity assay. *J. Thermoplast. Compos. Mater.* **36**, 2998–3020 (2023).

[68] Goy, R. C., Morais, S. T. & Assis, O. B. Evaluation of the antimicrobial activity of Chitosan and its quaternized derivative on E. coli and S. aureus growth. *Revista Brasileira De Farmacognosia*. **26**, 122–127 (2016).

[69] Varma, R., Vasudevan, S. & Extraction Characterization, and antimicrobial activity of Chitosan from horse mussel Modiolus modiolus. *ACS Omega*. **5**, 20224–20230 (2020). 32832775 10.1021/acsomega.0c01903 PMC7439375

[70] Chen, F. C., Huang, C. M., Yu, X. W. & Chen, Y. Y. Effect of nano zinc oxide on proliferation and toxicity of human gingival cells. *Hum. Exp. Toxicol.* **40**, S804–s813. 10.1177/09603271211058063 (2021). 34797187 10.1177/09603271211058063

[71] Pinto, E. P. et al. Influence of low and high glycerol concentrations on wettability and flexibility of Chitosan biofilms. *Quim. Nova*. **41**, 1109–1116 (2018).

[72] Rotta, J. et al. Parameters of color, transparency, water solubility, wettability and surface free energy of Chitosan/hydroxypropylmethylcellulose (HPMC) films plasticized with sorbitol. *Mater. Sci. Engineering: C*. **29**, 619–623 (2009).

[73] El-Hefian, E. A., Nasef, M. M. & Yahaya, A. H. The Preparation and characterization of Chitosan/poly (vinyl alcohol) blended films. *E-J. Chem.* **7**, 1212–1219 (2010).

[74] Choo, K., Ching, Y. C., Chuah, C. H., Julai, S. & Liou, N. S. Preparation and characterization of Polyvinyl alcohol-chitosan composite films reinforced with cellulose nanofiber. *Materials* **9**, 644 (2016). 28773763 10.3390/ma9080644 PMC5509094

[75] Sultan, M., Youssef, A. & Baseer, R. A. Fabrication of multifunctional ZnO@tannic acid nanoparticles embedded in chitosan and polyvinyl alcohol blend packaging film. *Sci. Rep.* **14**, 18533. 10.1038/s41598-024-68571-9 (2024). 39122764 10.1038/s41598-024-68571-9 PMC11316066

[76] Jayakumar, A., Radoor, S., Shin, G. H., Siengchin, S. & Kim, J. T. Active and intelligent packaging films based on PVA/Chitosan/Zinc oxide nanoparticles/sweet purple potato extract as pH sensing and antibacterial wraps. *Food Biosci.* **56**, 103432. 10.1016/j.fbio.2023.103432 (2023).

